# Frequency and consequences of the collection of already parasitized caterpillars by a potter wasp

**DOI:** 10.1038/s41598-020-65096-9

**Published:** 2020-05-26

**Authors:** Michal Segoli, Sarah Leduc, Fengqun Meng, Ishai Hoffmann, Miriam Kishinevsky, Tamir Rozenberg

**Affiliations:** 1 Mitrani Department of Desert Ecology, The Jacob Blaustein Institutes for Desert Research, Ben-Gurion University of the Negev, Sede Boqer Campus, Israel; 2 Department of Evolutionary and Environmental Biology, University of Haifa, Haifa, Israel

**Keywords:** Ecology, Evolution

## Abstract

Maladaptive behaviors reflecting a “bad” choice of habitat or resource have been widely documented; however, their persistence is often difficult to interpret. The potter wasp *Delta dimidiatipenne* constructs mud cells, in each of which it lays a single egg and places several caterpillars to feed its offspring. Preliminary observations indicated that a portion of these caterpillars were already parasitized and contained the offspring of the gregarious parasitoid *Copidosoma primulum*. As a result, the offspring of the potter wasp often failed to develop. To characterize the distribution, frequency and consequences of this intriguing phenomenon, we surveyed potter wasp nests throughout the Negev Desert. Evidence for parasitized caterpillars (mummies) was found in ~85% of the sampled sites, in ~20% of previous years’ nest cells and in ~70–80% of the same year’s cells. The survival and pupal mass of the potter wasp offspring were negatively associated with the presence and number of parasitized caterpillars inside the cells. We concluded that the collection of parasitized caterpillars by *D. dimidiantipenne* is frequent and costly. The persistence of this behavior may result from limited discrimination ability against parasitized prey by female potter wasps, or by their limited ability to exhibit choosiness under field conditions.

## Introduction

Under the assumptions of natural selection, animals are expected to evolve traits that increase their reproductive success in the environment. Nevertheless, maladaptive traits, *i.e*., traits that do not contribute and could even negatively affect the fitness of an organism, are often observed in nature^1^. In particular, maladaptive behaviors reflecting a “bad” choice of habitat, resource, mate, or oviposition sites have been widely documented. The persistence of such behaviors is often difficult to interpret; yet, it may have severe consequences for the survival of individuals, populations, and even species^2,3^.

Some maladaptive behaviors may represent a response to manipulations imposed by a different species. For example, many parasites induce their host to behave in a maladaptive way to enhance their own transmission^4,5^. Other maladaptive responses may be expressed as the result of rapid environmental changes that cause a mismatch between environmental cues and their adaptive value^6–8^. Such situations are often termed “ecological” or “evolutionary traps” as the organism is attracted to a novel habitat or resource with unexpected negative outcomes. For example, Albatrosses and other seabirds were shown to ingest and to feed their chicks with plastic objects that resemble their prey^9,10^. In addition, several parasitoid wasps were shown to lay eggs in an invasive host, unsuitable for their development^11,12^. However, maladaptive behaviors may persist even in the absence of a recent environmental change^13^. Moreover, some behaviors may entail deleterious outcomes to all interacting players. Such responses have received little attention, and the mechanisms maintaining their persistence are poorly understood. Here, we investigated the occurrence of such an extreme interaction, where all species involved seem to suffer a high cost.

The potter wasp *Delta dimidiatipenne*, Saussure (Hymenoptera, Vespidae, Eumeninae) collects caterpillars to feed its offspring. Females construct mud cells, in each of which they lay a single egg and place several paralyzed caterpillars. When the egg hatches, the juvenile completes its development while feeding on the “preserved meal” and eventually breaks out of the mud cell as an adult^14^. Preliminary observations (D. Gerling 1967, confirmed by M. Segoli 2017, unpublished data) revealed that a large proportion of potter wasp nest cells in a certain location (Havarim Wadi, Negev Desert, Israel) failed to develop and remained sealed. Closer examination indicated that some of the caterpillars placed by the potter wasps in these cells were already parasitized and contained the offspring of the gregarious parasitoid *Copidosoma primulum*, Mercet (Hymenoptera, Encyrtidae) inside their bodies. As a result, all players in the interaction perished—the caterpillars were consumed internally by *C. primulum* larvae (as evidenced by their distinct remains); the potter wasp offspring could not fully exploit the parasitized caterpillars and presumably starved to death (as evidenced by the absence of an emergence hole); and the *C. primulum* parasitoids completed their development, but could not break out of the mud and remained trapped in the sealed cell (as evidenced by their dead bodies).

This raises the following questions: why do potter wasps collect caterpillars parasitized by *C. primulum* in their nests, and how is this behavior maintained? This is clearly not the result of a manipulation by another species, as the outcome is costly to all players. It is also not likely to represent a novel interaction (and hence an ecological trap), as it was observed over 50 years ago (D. Gerling, personal communication), and presumably all interacting species are native to the area (see Methods section). Models predict that maladaptive behaviors could persist depending on the portion of individuals in the population exposed to the low quality habitat or resource, the severity of the outcome of exposure, and the ability of the organism to evolve a corrective behavior^15–17^. On this basis, it can be hypothesized that the interaction between potter wasps and caterpillars parasitized by *C. primulum* is either not frequent enough or not costly enough (*e.g*., not always fatal) to induce a strong selection pressure. Alternatively, constraints may prevent the potter wasps from evolving or exhibiting discrimination against parasitized prey.

Here, we took a first step in addressing these hypotheses by characterizing the geographical distribution and frequency of the interaction between potter wasps and caterpillars parasitized by *C. primulum*, and by quantifying some of its costs and consequences. For this, we surveyed potter wasp nests in multiple locations throughout the Negev Desert in Israel, quantified the proportion of cells containing caterpillars parasitized by *C. primulum*, and related their occurrence to cell fate (*i.e*., developmental failure and body size of the potter wasp offspring). In addition, we collected caterpillars from nearby vegetation to determine the natural parasitism rate by *C. primulum*, and we observed potter females during nest construction. We hypothesized that if the time invested in caterpillar collection is substantial, and if the parasitism rate is high, females may be limited in their ability to evolve or exhibit choosiness under natural conditions.

## Methods

### Study species

#### *Delta dimidiatipenne*

The caterpillar hunting wasp *D. dimidiatipenne* is named for its habit of collecting caterpillars to feed its offspring. Females construct nests of ~20 cells during the spring (March–May). Adult wasps are around 25 mm long and feed on floral nectar. The species’ geographical distribution is wide-ranging, spanning from northwest Africa, Egypt and Somalia, throughout the Middle East, and east to India and Nepal. In Israel, its distribution ranges from the center to the south of the country^14^. Preliminary observations suggested that potter wasp females in the Negev Desert often collect caterpillars of the family Noctuidae. In particular, caterpillars of the native species *Heliothis nubigera*^18^ are the most common prey item found both in potter wasp nests and on nearby *Zygophyllum dumosum* shrubs, where potter wasps were observed to forage (M. Segoli and T. Rozenberg, personal observations).

#### *Copidosoma primulum*

Parasitoids of the genus *Copidosoma* are polyembryonic, *i.e*., each wasp egg proliferates to produce a clone of genetically identical embryos. Females parasitize the egg stage of their host (mostly lepidopteran). The host larva hatches and develops to its final instar. During this period, the *Copidosoma* egg proliferates clonally to produce numerous genetically identical embryos inside the host^19–22^. Embryos develop into larvae that consume the host internally and eventually pupate within the remnant host cuticle to form a “mummy”. Adult wasps (typically 1–2 mm in size) emerge from the mummy around two weeks later. Importantly for this study, the mummies have a distinct appearance that is easily identifiable even following wasp emergence. Parasitoids found in potter wasp nests in the Negev are of the species *C. primulum*. Although to date, there has been no formal description of this species from Israel, it is known from similar environments in Europe, Africa, and Central Asia^23^, and hence is likely to be native to this area. In the Negev Desert, *C. primulum* were found to develop in moth caterpillars of the family Noctuidae (Lepidoptera) and specifically on the common native species *Heliothis nubigera*.

### Survey of previous-years nests

**Figure 1:**
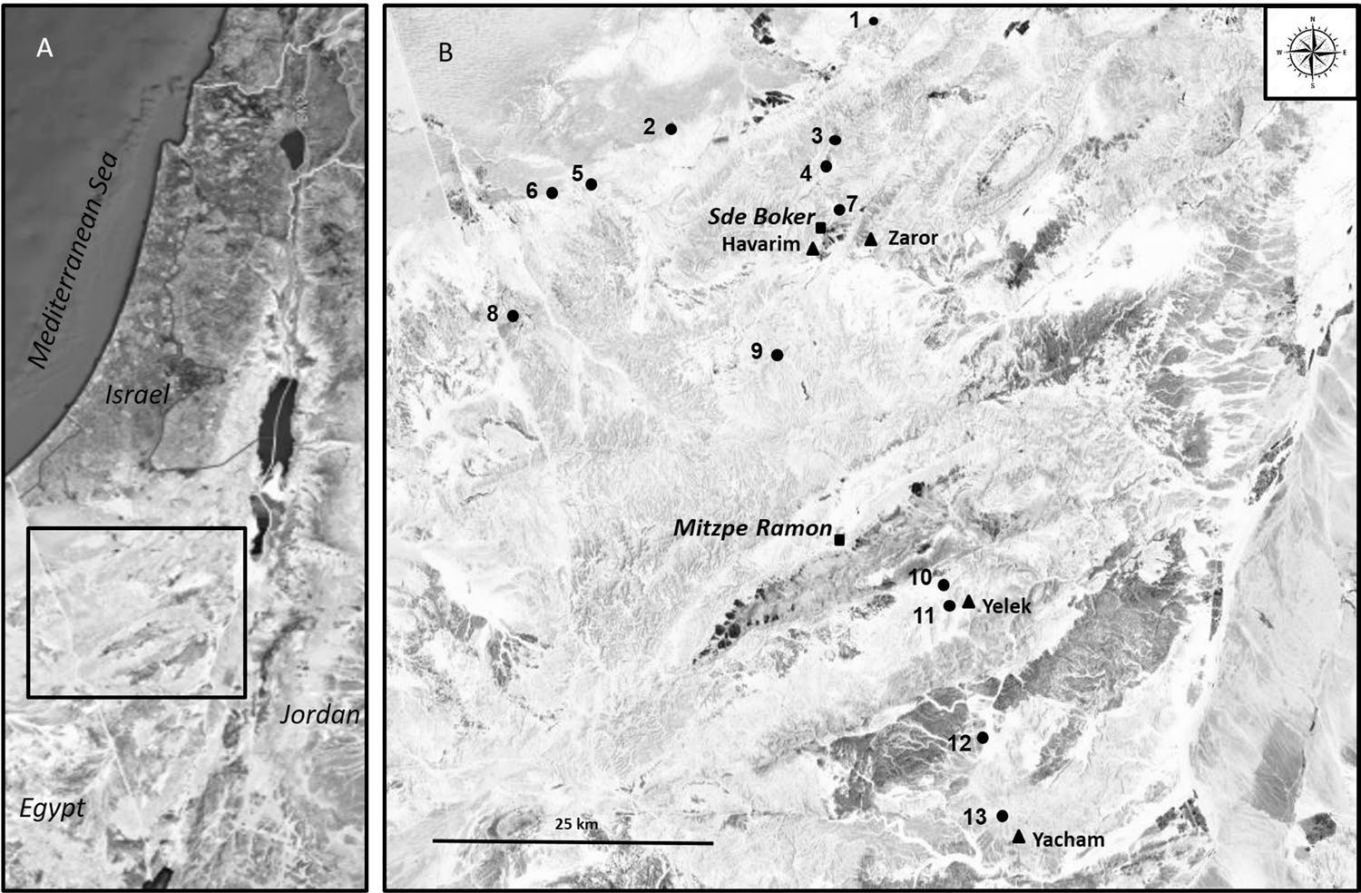
Map of sampling sites in the Negev Desert during the potter wasp nest surveys, 2017–2018. (**A**) Map of Israel; (**B**) enlarged section of the Negev Desert. Circles represent sites of the previous-years nests survey, triangles represent sites of the same-year nests survey and squares represent the main villages in the area. Figure was created using Microsoft PowerPoint 2010 by modifying image from Google Maps (Map data ^©^2019, Google, Mapa GISrael).

To obtain general information on the geographical distribution and frequency of the collection of parasitized caterpillars, we surveyed potter wasp nests of previous years (age unknown) during January 2018, in 13 sites throughout the Negev Desert (Fig. 1). Potter wasp nests (*i.e*., cell clusters) were found mostly in water passages under vehicle roads or under bridges. *D. dimidiatipenne* nests were identified based on their size and shape, although occasionally we might have sampled the nests of other potter wasp species that occur in the area (*e.g., Katamenes dimidiativentris*). In each site, we collected the content of up to 10 cells, from each of up to 10 nests (depending on availability) for a total of 505 cells. Evidence for *C. primulum* presence (*i.e*., dead adult wasps or remains of parasitized caterpillar mummies) was documented. While this wide survey allowed us to obtain some basic information on the distribution and frequency of this interaction in the Negev Desert, the poor condition of the nests (some of which were partially disintegrated or contained secondary residences such as spiders or beetles) might have caused biases in our estimations (*e.g*., some evidence of *C. primulum* might have been lost, or only preserved in certain cells). Hence, to achieve more accurate estimations and to relate cell fate to the presence of caterpillars parasitized by *C. primulum*, we conducted a second survey of newly constructed *D. dimidiatipenne* nests (see below).

### Survey of same-year nests during development and subsequent emergence

**Figure 2:**
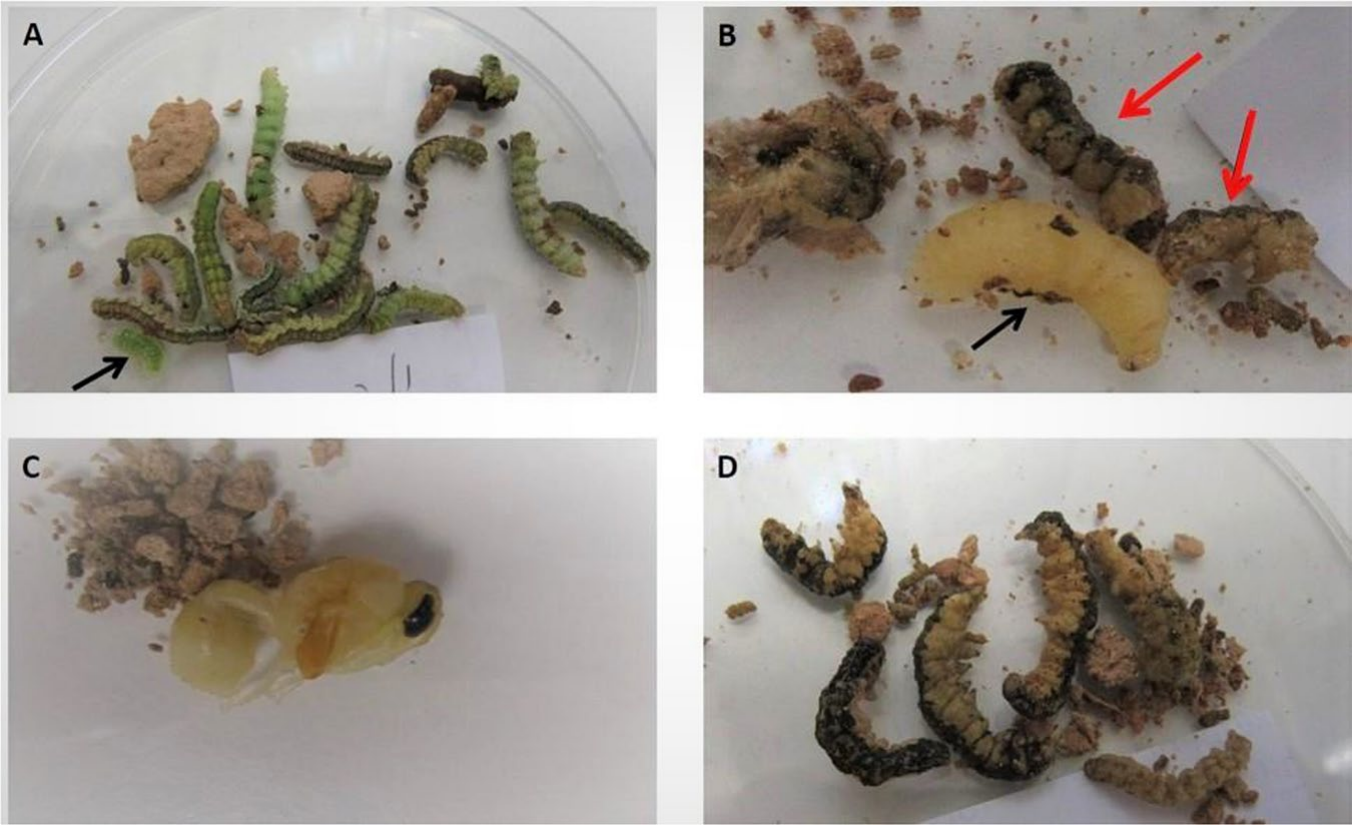
Typical findings in potter wasp cells sampled during wasp development: (**A**) a live young potter wasp larva (black arrow) with recently collected caterpillars; (**B**) a potter wasp larva at a later stage of development (black arrow), which survived despite the remains of caterpillars parasitized by *C. primulum* (red arrows); (**C**) a potter wasp pupa and no evidence for *C. primulum*; (**D**) a nest full of parasitized caterpillars (potter wasp offspring presumably dead).

This survey was conducted in four sites nearby water holes (Fig. 1), during the spring (Apr–May) of 2018. Newly constructed nests (of the same year) were identified via direct observations of active *D. dimidiatipenne* females during nest building, or according to nest appearance (made of smooth fresh mud). We documented the contents of 69 cells of 10 nests from these sites during potter wasp development. Since potter wasp females add a few cells to their nest every day, potter wasp offspring in these nests were at various developmental stages (mostly larval or pupal). Evidence for the presence of *C. primulum* in the cells was documented; however, the initial number of healthy caterpillars placed in each cell was often difficult to determine since some could have already been eaten by the potter wasp larvae. Cell content was further characterized as having either a live potter wasp larva, a dead or absent (assumed dead) potter wasp larva, or a potter wasp pupa (see Fig. 2 for examples of typical cell contents). While we could not predict what would have been the fate of still living larvae if not collected, the occurrence of dead or no larvae indicated that the potter wasp offspring did not survive, while the occurrence of pupae indicated that the potter wasp offspring were able to complete their development. These data allowed us to quantify the proportion of *D. dimidiatipenne* cells with evidence for caterpillars parasitized by *C. primulum* more accurately than in the first survey, and to relate the occurrence of parasitized caterpillars with potter wasp offspring survival, developmental stage, and pupal mass.

In addition, five newly constructed nests (*N* = 82 cells in total) were kept untouched until the end of the season. These nests were sampled following potter wasp emergence, allowing us again to quantify the proportion of cells that contained caterpillars parasitized by *C. primulum*, and also to relate their presence to cell failure (as indicated by the lack of  emergence hole).

### Behavioral observations

To characterize the investment of potter wasps in nest building, we directly observed nine potter wasp females during nest building in the sites surveyed for the same-year nests (overall 10 observations from the beginning till the end of cell construction). We quantified the number and duration of each component of the cell building process. In particular, we were interested in the relative investment in caterpillar collection *vs.* other components of the nest building.

### Proportion of parasitized caterpillars on natural vegetation

To determine the parasitism rate by *Copidosoma* on caterpillars in the field, we collected *Heliothis* caterpillars from *Zygophyllum dumosum* shrubs near a potter wasp activity zone in the Havarim Wadi in March 2018 (*N* = 15) and dissected them under the microscope to search for parasitoid larvae. An additional sample was taken in Yacham in March 2019 (*N* = 39) and caterpillars were either dissected (*N* = 21) or reared till moth or *Copidosoma* pupation (*N* = 18).

### Statistical analyses

We calculated the proportion of sites, nests, and cells with evidence for *C. primulum* in the survey of previous-years nests. The effects of the presence and number of caterpillars parasitized by *C. primulum* in a cell (1, 2, 3, 4, or more than 4), on potter wasp offspring survival and pupation success in same-year nests were tested via a nominal logistic regression. The effect of *C. primulum* presence on potter wasp offspring pupal mass was tested using a one-tailed t-test. The choice of this test was based on our assumption that parasitized caterpillars cannot be readily consumed by the potter wasp larvae, and hence their presence in the cell should lead to lower pupal mass. Due to the small number of cells with parasites other than *C. primulum* and their unknown role (see below), we did not examine their effect on cell fate. Statistical analyses were performed using JMP version 13.0.0 (SAS Institute Inc., Cary, North Carolina, USA).

## Results

### Survey of previous-years nests

Altogether we sampled 505 cells from 113 clusters in 13 sites. Evidence of *C. primulum* presence was found in 11 out of the 13 sampled sites, in 28% ± 7% of the nest clusters per site, and in 19% ± 5% (mean ± SE) of the cells per site (see Table 1). The overall distribution of this interaction ranged throughout the Negev Desert (Fig. 1).

**Table 1:** Number of potter wasp nests and nest cells sampled during a survey of previous-years nests, and the proportion of nests and cells with evidence for *C. primulum* (dead wasps or remains of parasitized caterpillar mummies).

Site	Number of nests sampled	Prop nests with *C. primulum*	Number of cells sampled	Prop cells with *C. primulum*
1	7	0.14	24	0.17
2	10	0	49	0
3	10	0.30	29	0.34
4	4	0.25	13	0.38
5	10	0.10	48	0.10
6	10	0.10	50	0.02
7	10	0.40	43	0.26
8	10	0.20	42	0.10
9	10	0.70	46	0.20
10	6	0.33	25	0.12
11	10	0.80	35	0.54
12	10	0.40	42	0.24
13	6	0	30	0
Mean ± SE	8.69 ± 0.59	0.28 ± 0.07	36.62 ± 3.21	0.19 ± 0.05

Other than *C. primulum*, some cells in our survey contained parasites and parasitoids of either the potter wasp or caterpillars or both. Nest parasitoids included *Melittobia acasta* (Hymenoptera, Eulophidae), a new finding for Israel, and cuckoo wasps (Hymenoptera, Chrysididae). One unidentified species of ichneumonid (Hymenoptera, Ichneumonidae) and braconid wasps (Hymenoptera, Braconidae) are probably lepidopteran caterpillar parasitoids, but this was not verified. A species of the genus *Monodontomerus* (Hymenoptera, Torymidae), bombyliid flies (Diptera, Bombyliidae), and tachinid flies (Diptera, Tachinidae) were also found in the nests, but their host species could not be determined. Finally, some cells contained spiders, beetles, and bees that were probably secondary residents in the nests.

### Survey of same-year nests during development

**Figure 3:**
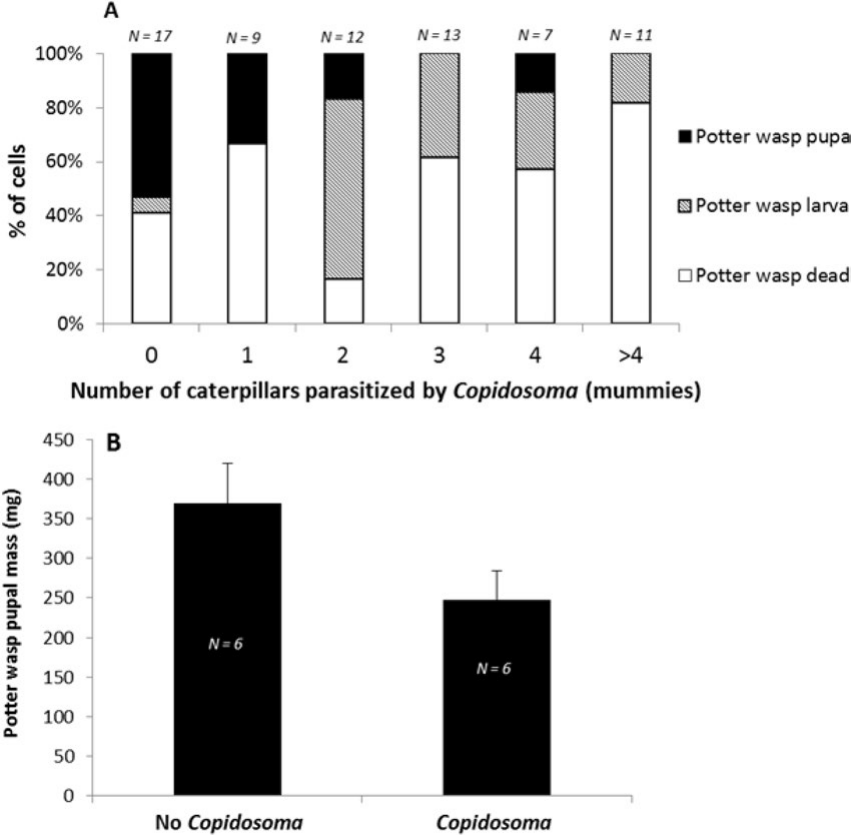
(**A**) Percentage of cells that contained: dead or absent potter wasp larva ; live potter wasp larva ; or potter wasp pupa in relation to the number of caterpillars parasitized by *C. primulum* (mummies) found in the cell. (**B**) Body mass of potter wasp offspring that pupated in cells containing evidence for *C. primulum* (right column, N = 6), or that did not contain *C. primulum* (left column, N = 6).

We found evidence of *C. primulum* presence in all four sites sampled, in 9 out of the 10 nest clusters, and in 71% of the cells (*N* = 69 pooled across sites). Cells with evidence for *C. primulum* were more likely to contain dead or no potter wasp larvae, and were less likely to contain potter wasp offspring that reached the pupal stage (Fig. 3A; nominal logistic regression, *df* = 2, χ^2^ = 11.36, *P* = 0.003). Moreover, potter wasp larva survival was further reduced as the number of parasitized caterpillar mummies found in a cell increased (Fig. 3A; nominal logistic regression, *df* = 8, χ^2^ = 24.38, *P* = 0.002). Finally, potter wasp pupae from cells with *C. primulum* had a lower body mass than those from cells without *C. primulum* (Fig. 3B; one-tailed t-test, t_1_ = 1.95 *P* = 0.04, *N* = 12). Hence, the presence of *C. primulum* seemed to have had a negative effect on potter wasp survival rate and body mass, but was not necessarily fatal. In accordance, we found evidence for parasitized mummies that were partially consumed—indicating that the potter wasp larvae could potentially feed on parasitized caterpillars prior to *C. primulum* pupation.

Other than *C. primulum*, several cells contained other potential parasites including a cuckoo wasp (Chrysididae) in one cell, torymid wasps in three cells, fly pupae in five cells, and a braconid wasp in one cell. None of these cells contained live potter wasp offspring.

### Survey of same-year nests following wasp emergence

**Figure 4:**
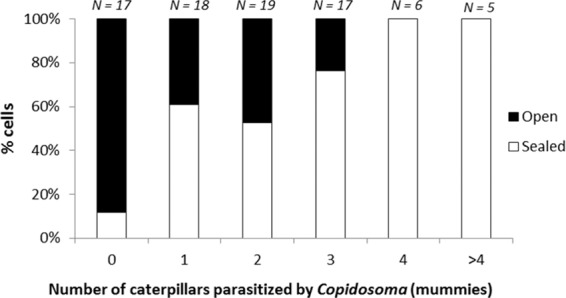
Percentage of cells that were either sealed or opened—presumably by the potter wasp offspring—in relation to the number of caterpillars parasitized by *C. primulum* (mummies) found in the cell.

We found evidence of *C. primulum* presence in 79% of the same-year cells that were sampled at the end of the season (*N* = 82 cells). The presence of *C. primulum* was positively related with cell failure (Fig. 4; nominal logistic regression, *df* = 1, χ^2^ = 21.87, *P* < 0.001). Moreover, the probability of cell failure further increased with the number of parasitized caterpillar mummies per cell (nominal logistic regression, *df* = 5, χ^2^ = 12.07, *P* = 0.034). This again suggests that the presence of caterpillars parasitized by *C. primulum* reduces potter wasp developmental success, but does not entirely eliminate it.

Other than *C. primulum*, we found evidence for fly parasites in 13 cells, for braconid wasps in four cells, for torymid wasps in one cell, and for a cuckoo wasp in one cell.

### Behavioral observations

**Figure 5:**
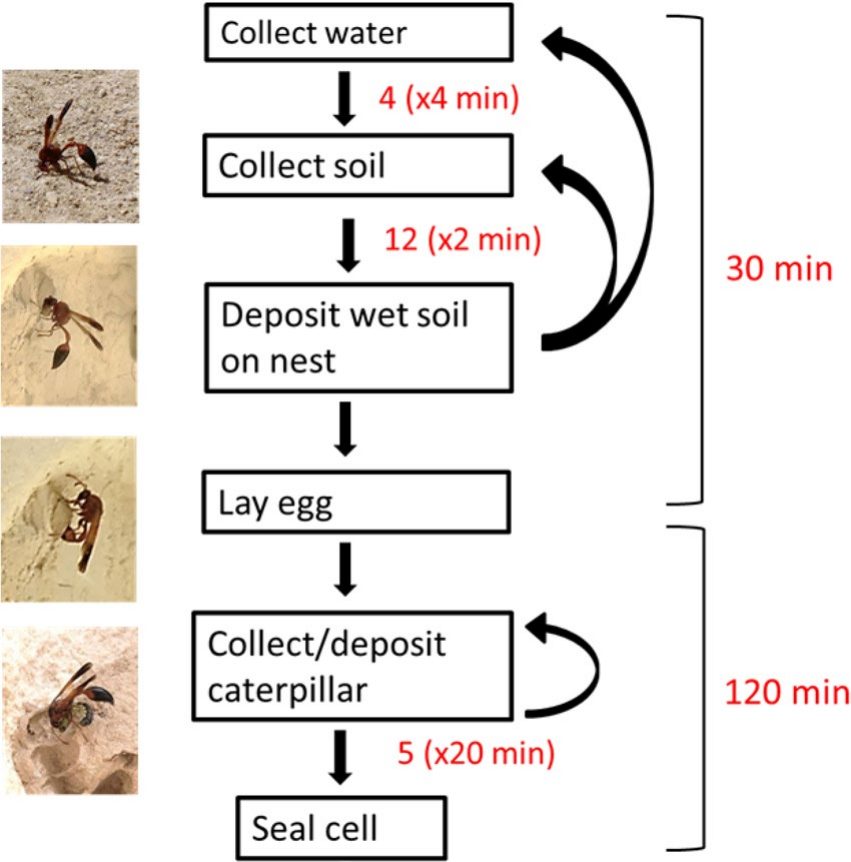
Ethogram of cell construction behavior and accompanying pictures demonstrating the different stages. Numbers on each arrow represent the estimated number of times a certain action was repeated and its mean duration (in parentheses).

Direct observations of females during cell construction (N = 10) revealed that the construction of a single cell included 4 ± 0.5, 2–6 (Mean ± SE, Range) excursions for collecting water, taking 4.3 ± 0.8, 1.4–8 min each, and 12.3 ± 1.5, 9–24 excursions to collect soil, taking 1.6 ± 0.2, 1–2.5 min each, which was then deposited on the nest. At the completion of the pot structure, the female laid a single egg inside the cell. The female then disappeared to forage for caterpillars that were then placed in each cell for a total of 5.2 ± 0.3, 3–7 caterpillars; each excursion for a single caterpillar took 19.5 ± 3.6, 9–39 min. The female then sealed the cell and deposited additional soil on the cell and on adjacent cells, presumably to protect it from parasites^24^. Altogether, cell construction took 34.4 ± 4.5, 21–57 min till oviposition, and an additional 120.2 ± 18.4, 59–218 min till cell provisioning was completed, and the cell was sealed. Hence, caterpillar collection constituted a major portion of the time dedicated to nest building. An ethogram of the nest building behavior is depicted in Fig. 5.

### Proportion of parasitized caterpillars on natural vegetation

Six out of 15 (40%) of the caterpillars collected in Havarim, contained *Copidosoma* embryos or larvae, which were easily identifiable^19^. In addition, one caterpillar contained a single larva of an unknown solitary parasitoid. 12 out of 39 (31%) of the caterpillars collected in Yacham, contained *Copidosoma* larvae while dissected or produced *Copidosoma* following rearing. In addition, three caterpillars produced other unidentified parasitoids.

## Discussion

The collection of parasitized caterpillars by females of the potter wasp *D. dimidiatipenne* seems maladaptive as females invest time and energy in building and provisioning cells with resources that ultimately will not (or will hardly) serve their offspring. The results of our field survey suggest that this interaction is not rare or anecdotal, and that it entails a high cost in terms of reduced survival and potential fitness of the potter wasp offspring. Hence, it is likely to impose a strong selection on the potter wasps.

Estimations of the proportion of cells with parasitized caterpillars varied widely among the sampled locations and was generally lower when surveying previous-years nests (~20%), than when surveying same-year nests (~70–80%). However, even if we consider the lower estimation range, the proportion of the population that is likely to be affected and the loss of reproductive success to the potter wasps, seems substantial. Moreover, this phenomenon was found to be widely distributed throughout the Negev Desert and, hence, cannot be considered as a local sink. Given the presumed large overlap in the distribution of the studied species, it is likely to be even more widely distributed than documented here. Finally, similar interactions may potentially occur involving other potter wasp species that collect similar prey or whose prey also suffer high parasitism rates. Indeed, in several anecdotal reports, the authors documented the occasional occurrence of parasitized prey or prey-parasites^25–29^ in potter wasp nests. However, the distribution and consequences of such interactions were not examined further. We suggest that while the phenomenon by itself is not unique, the high frequencies and consequences reported here are probably exceptional.

Despite the implied reduced survival of *D. dimidiatipenne* potter wasp offspring, the presence of parasitized caterpillars was not always fatal. This could be explained by the simultaneous occurrence of non-parasitized caterpillars inside the cells, sufficient for the potter offspring’s nourishment. In addition, although potter wasp offspring are not likely to feed on sclerotized pupae or adult *C. primulum* parasitoids, they may still be able to feed to some extent on parasitized caterpillars at earlier developmental stages, as implied by the presence of partially consumed mummies. Hence, parasitized caterpillars could still occasionally provide a limited amount of food to the potter wasp offspring, depending on the timing of potter wasp development relative to *C. primulum* pupation. Our observations suggest that this window of opportunity is short—as we have often observed potter wasp larvae cohabiting with already mummified caterpillars.

Under the abovementioned scenarios, the cost may sometimes be manifested in the development of smaller potter wasp offspring, rather than in their mortality. Indeed, we found that potter wasp offspring that reached pupation in cells with parasitized caterpillars had a lower body mass, suggesting that they will develop into smaller adults. The consequences of smaller body size for *D. dimidiatipenne* adult reproductive success have not yet been explored; however, body size is known to be related to adult fitness in many insects^30^. Specifically in potter and other solitary nest-provisioning wasps, larger females were shown to live longer, provision more offspring, and collect more and heavier food items in their nests than smaller females^31–33^. Hence, even in cases in which the potter wasp offspring survive to complete their development, the occurrence of parasitized caterpillars in the cell is likely to compromise their fitness.

Given the high frequency and high implied costs, the question as to why *D. dimidiatipenne* potter wasps collect parasitized caterpillars becomes even more intriguing. One possibility is that certain constraints prevent potter wasps from evolving or exhibiting discrimination against parasitized caterpillars. Many parasitoid wasps are able to discriminate against already parasitized hosts, on which the survival and development of their offspring are compromised^34,35^. Although selection in relation to prey species is probably common in potter wasps^36–39^, discrimination in relation to prey parasitism status has rarely been reported. In one case, females of the species *Euodynerus foraminatus* were observed to inspect and evacuate caterpillars from their nests^31^. This was speculated to be a mechanism to eliminate parasites of the wasps or parasitized caterpillars; however, this interpretation has not been confirmed. Hence, the ability of potter wasps, in general, and of *D. dimidiatipenne*, specifically, to identify already parasitized prey is yet unknown.

Another option is that the high costs of examining prey items and discriminating while foraging in the field may overcome their potential advantages^40^. This may be especially true in light of potential environmental risks such as predation and parasitism. Indeed, potter wasps are known to be attacked by birds while foraging and to suffer from parasitism in their nest^25,28,37,41,42^. Our observations of *D. dimidiatipenne* females during nest building revealed that the largest portion of their time was devoted to searching for caterpillars to provision their nest. In addition, we observed potential parasites (flies and wasps) visiting cells while the potter wasp was absent (M. Segoli and T. Rozenberg, personal observation), as well as developing inside the nest cells. Such environmental risks may hinder females from evolving or exhibiting choosiness under natural conditions.

The acceptance of a low-quality resource may also depend on the availability of alternatives. Both theoretical models and empirical evidence suggest that individuals are more likely to accept poor quality food items or hosts if the availability of high quality resources in the environment is limited^43^. For example, parasitoid females are more likely to accept an already parasitized host, if they had previously experienced encounters with such low quality hosts^35,44,45^. Our data suggest that the parasitism rate by *Copidosoma* on caterpillars in the field was not negligible. Hence, even if potter wasp females have the potential ability to discriminate against parasitized caterpillars, they may not exhibit such choosiness if they frequently encounter already parasitized caterpillars in the field.

Another intriguing possibility is that innate biases make the parasitized caterpillars even more attractive to the foraging potter wasp females. For example, it was previously shown that caterpillars parasitized by *Copidosoma* spp. feed for longer durations and reach a higher mass than non-parasitized ones^19,20,46^. In addition, preference for larger prey items was demonstrated in several species of predatory wasps, at least within the limitation of wasp size^47–50^. Hence, *D. dimidiatipenne* potter wasp females may exhibit a preference for parasitized caterpillars due to their higher mass, despite their eventual poor suitability for feeding their offspring. Finally, parasitism by *C. primulum* may induce the caterpillars to behave in a way that makes them more exposed or vulnerable to potter wasp females, *e.g*., due to their different feeding habits or compromised immune system. These possibilities should be further explored.

Responses at the population level should also be considered. Despite the high costs from the collection of parasitized prey, *D. dimidiatipenne* populations in the Negev Desert seem to remain viable. This may suggest that females are able to partially compensate for these costs. Compensation via collecting other prey types is not likely as *H. nubigera* is the most common prey species in the nests. This may reflect its high density in the environment, possibly enhanced by the wasp conditioning on this prey type, as demonstrated in other wasp species^51^. Instead, potter wasps probably compensate by their ability to construct many cells during their lifetime. In fact, many solitary and parasitoid wasps suffer high rates of developmental failure due to varied causes, including host unsuitability and interference with other parasitoids^52–54^. Such cases of developmental failure often still cause the death of the host/prey and hence may have subsequent negative effect on their populations^53^. Although in the case described here, already parasitized caterpillars have minimal survival prospects whether or not they are collected to the nests, some cells that fail to develop may also contain healthy caterpillars. Hence, studying the population dynamics and feedback mechanisms between these three interacting species could shed further light on the occurrence and persistence of this phenomenon.

In conclusion, we have demonstrated high frequency and substantial costs for the collection of parasitized caterpillars by *D. dimidiatipenne* females. Additional observations and experimentations are required in order to further determine the spatial and temporal variations in the parasitism rate and population densities in the field; as well as to test whether parasitism by *C. primulum* induces a change in caterpillar attractiveness or susceptibility to potter wasp females. Such data will shed light on the mechanisms for the maintenance of this specific interaction, as well as on the persistence of maladaptive behaviors in nature, in general.

## Data Availability

The datasets generated during and/or analyzed during the current study are available from the corresponding author on reasonable request.
